# Ganglioside GM3 Up-Regulate Chondrogenic Differentiation by Transform Growth Factor Receptors

**DOI:** 10.3390/ijms21061967

**Published:** 2020-03-13

**Authors:** Jae-Sung Ryu, Sang Young Seo, Eun-Jeong Jeong, Jong-Yeup Kim, Yong-Gon Koh, Yong Il Kim, Young-Kug Choo

**Affiliations:** 1 Department of Otorhinolaryngology-Head and Neck Surgery, College of Medicine, Konyang University, Daejeon 35365, Korea; jsryu@kyuh.ac.kr (J.-S.R.); jykim@kyuh.ac.kr (J.-Y.K.); 2 Department of Biomedical Informatics, College of Medicine, Konyang University, Daejeon 35365, Korea; 3 Department of Biological Science, College of Natural Sciences, Wonkwang University, Iksan 54538, Korea; tjtkddud463@naver.com (S.Y.S.); ej0314@kribb.re.kr (E.-J.J.); 4 Biotherapeutics Translational Research Center, Korea Research Institute of Bioscience and Biotechnology, Daejeon 34141, Korea; 5 Center for Stem Cell & Arthritis Research, Department of Orthopaedic Surgery, Yonsei Sarang Hospital, Seoul 06698, Korea; osygkoh@gmail.com; 6 Department of Stem Cell Research, TJC Life Research and Development Center, TJC Life, Seoul 08512, Korea

**Keywords:** human synovial-derived mesenchymal stem cells, chondrogenic aggregates differentiation, gangliosides

## Abstract

Mesenchymal stem cells, also known as multipotent stromal progenitor cells, can differentiate into cells of mesodermal lineage. Gangliosides are sialic acid-conjugated glycosphingolipids that are believed to regulate cell differentiation and several signaling molecules. These molecules are localized in glycosphingolipid-enriched microdomains on the cell surface and are regulated by glycosphingolipid composition. Transforming growth factor-beta (TGF-β) signaling plays a critical role in chondrogenic differentiation. However, the role of gangliosides in chondrogenesis is not understood. In this study, the relationship between the ganglioside GM3 and TGF-β activation, during chondrogenic differentiation, was investigated using an aggregate culture of human synovial membrane-derived mesenchymal stem cells. We showed that the gangliosides GM3 and GD3 were expressed after the chondrogenic differentiation of hSMSC aggregates. To test whether GM3 affected the chondrogenic differentiation of hSMSC aggregates, we used GM3 treatment during chondrogenic differentiation. The results showed that the group treated with 5 μM GM3 had higher expression of chondrogenic specific markers, increased toluidine blue, and safranin O staining, and increased accumulation of glycosaminoglycans compared with the untreated group. Furthermore, GM3 treatment enhanced TGF-β signaling via SMAD 2/3 during the chondrogenic differentiation of hSMSC aggregates. Taken together, our results suggested that GM3 may be useful in developing therapeutic agents for cell-based articular cartilage regeneration in articular cartilage disease.

## 1. Introduction

Mesenchymal stem cells (MSCs), also referred to as multipotent stromal progenitor cells, can be differentiated into cells of mesodermal lineage, such as chondrocytes, osteoblasts, and adipocytes, making them an interesting candidate in regenerative medicine [1,2]. Notably, synovium-derived mesenchymal stem cells (SMSCs) have a remarkable capacity for self-renewal and multipotency with common surface epitopes [3]. Moreover, SMSCs have been shown to have the greatest chondrogenic potential, owing to the presence of uridine diphosphoglucose dehydrogenase activity and expression of cartilage oligomeric matrix protein (COMP) and glycosaminoglycans (GAG) [4,5,6,7]. Therefore, many researchers have suggested that SMSCs offer a new strategy for clinical applications, such as cell-based cartilage regeneration.

Gangliosides are complex glycosphingolipids that contain one or more sialic acid residues and are the major components of cytoplasmic membranes [8]. Different cell types harbor diverse types of gangliosides that are involved in various biological processes, such as apoptosis, cell proliferation and differentiation, cell-surface interactions, and transmembrane signaling [9,10,11]. Numerous studies have confirmed that the pattern and level of ganglioside expression are subject to developmental and cell-type specific regulation [12,13]. Moreover, results from our recent studies have suggested that the expression of gangliosides is closely related to the differentiation of stem cells in vitro [14,15,16]. Interestingly, GM3 has been shown to regulate tyrosine phosphorylation of growth factor receptors [17,18,19].

Members of the transforming growth factor-beta (TGF-β) superfamily, TGF-β1, 2, and 3 play a critical role in proliferation [20]. TGF-β was shown to efficiently up-regulate various molecules associated with pre-chondrogenic condensation and chondrogenic gene expression, thereby inducing chondrogenic differentiation [21,22,23]. The TGF-β related signal transduction includes initial internalization via serine/threonine phosphorylation of TGF-β receptor (TGF-βR) after pairing of TGF-βR1 and TGF-βR2 on the cell surface [24]. Phosphorylated TGF-βR directly activates the downstream mediator receptor-activated SMAD, including SMAD2 and SMAD3 [24,25,26].

In this study, we investigated whether human SMSCs (hSMSCs) could differentiate into chondrocyte and the role of the ganglioside, GM3, in chondrogenic differentiation of hSMSCs.

## 2. Results

### 2.1. GM3 and GD3 Expression in the Chondrogenic Differentiation of hSMSC Aggregates

**Figure 1:**
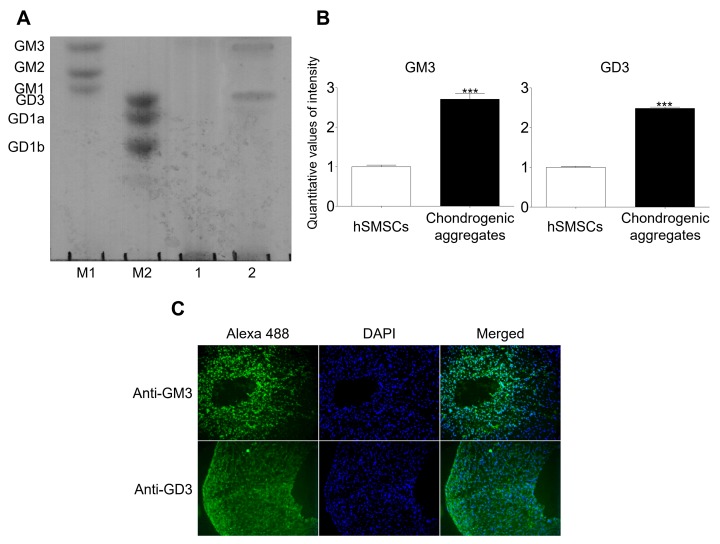
Analysis of ganglioside expression. (**A**) High-performance thin-layer chromatography (HPTLC) analysis of ganglioside expression in human synovial-derived mesenchymal stem cells (hSMSCs) and chondrogenically differentiated aggregates. M1 and M2, ganglioside standard markers; (1), human synovial-derived mesenchymal stem cells (hSMSCs); (2), Chondrogenic differentiation of aggregates. (**B**) Quantitative analysis of ganglioside expression in hSMSCs and chondrogenic aggregates differentiation. The quantitative values generated using the densitometry program (Image J) represent the mean values obtained from three separate experiments. *** *p* <0.001 compared with the hSMSCs. (**C**) Immunofluorescence analysis of GM3 and GD3 after chondrogenic differentiation of aggregates. GM3 and GD3 expression (Alexa 488; green) were detected after chondrogenic aggregates differentiation.

hSMSCs were characterized morphologically (fibroblastic morphology), evaluated for the presence of mesenchymal markers (CD44, CD73, CD90, and CD105), and assessed for mesodermal differentiation into chondrocytes, osteoblasts, and adipocytes (Supplementary Figure S1). Characterized hSMSCs were differentiated into chondrocytes using the aggregation culture system and showed chondrocyte marker expression upon differentiation (Supplementary Figure S2). Ganglioside expression patterns were analyzed by HPTLC after chondrogenic differentiation was induced in the aggregates (Figure 1). No gangliosides were detected in hSMCs; however, GM3 and GD3 were detected in aggregates with chondrogenic differentiation. Moreover, immunofluorescence analysis showed that GM3 and GD3 were expressed in aggregates with chondrogenic differentiation. Thus, we speculated that GM3 or GD3 might play a role in the chondrogenic differentiation of *hSMSC* aggregates.

### 2.2. GM3 Enhanced Chondrogenic Differentiation of hSMSC Aggregates

**Figure 2:**
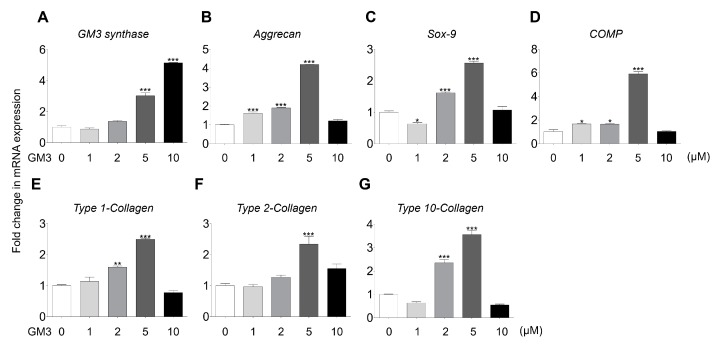
Comparison of chondrogenic specific markers expression after the chondrogenic differentiation of aggregates. GM3 (0, 1, 2, 5, and 10 μM) was used for treatment during the chondrogenic differentiation of hSMSC aggregates. (**A**) *GM3 synthase,* (**B**) *aggrecan,* (**C**) *SOX-9,* (**D**) *cartilage oligomeric matrix protein (COMP)*, (**E**) *type 1-Collagen*, (**F**) *type 2-collagen*, and (**G**) *type 10-collagen* expression were compared by qPCR. mRNA expression levels were normalized to the housekeeping gene β-actin. The values shown are the mean ± SEM from three independent experiments. * *p* < 0.05, ** *p* < 0.01 and *** *p* < 0.001 compared with the 0 μM GM3 treated group.

Several studies have demonstrated that GM3 improved differentiation of various cell types, but not the chondrogenic differentiation of *hSMSC* aggregates. Here, we studied the effect of GM3 treatment on the chondrogenic differentiation of hSMSC aggregates. To evaluate GM3 cytotoxicity, hSMSCs were cultured with various concentrations of GM3 (Supplementary Figure S3). The mean value of cell viability did not significantly differ from that of the 0 μM GM3 treated group during 7 d. Interestingly, tissue weight of chondrogenically differentiated hSMSC aggregates was seen to significantly increase in the 2 and 5 μM GM3 treated groups (0 μM, 7.67 ± 0.90 mg; 1 μM, 8.33 ± 0.25 mg; 2 μM, 10.13 ± 0.45 mg; 5 μM, 11.37 ± 0.72 mg; and 10 μM, 9.13 ± 0.47 mg) (Supplementary Figure S4). Furthermore, GM3 synthase expression increased in the 5 and 10 μM GM3 treated groups, and the expression of chondrogenic specific markers, such as *aggrecan*, 3.00 ± 0.02 folds; *SOX-9*, 2.85 ± 0.55 folds; *COMP*, 5.92 ± 0.26 folds; *type 1 collagen*, 2.50 ± 0.05 folds; *type 2 collagen*, 2.55 ± 0.19 folds; and *type 10 collagen*, 3.56 ± 0.31 folds, also significantly increased in the 5 μM GM3 treated group compared with that in the 0 μM treated group (Figure 2).

**Figure 3:**
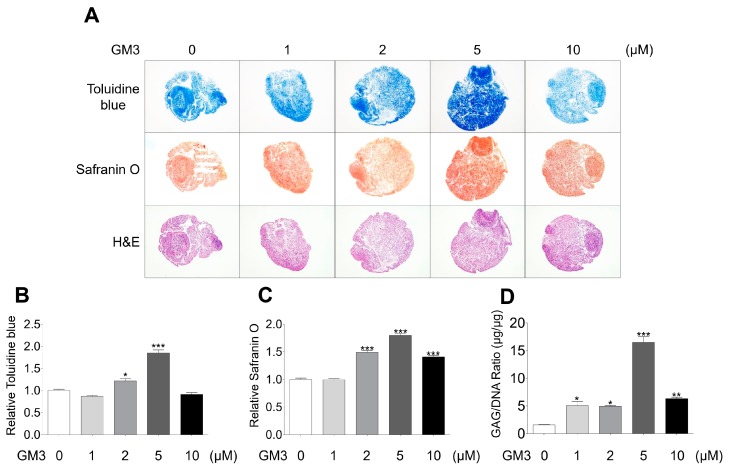
Histological and biochemical analysis of chondrogenically differentiated aggregates. (**A**) Chondrogenically differentiated aggregates from all groups were stained with toluidine blue, safranin O, and hematoxylin and eosin. Relative (**B**) toluidine blue and (**C**) safranin O intensity after chondrogenic differentiation. The values shown are the mean ± SEM from three independent experiments. * *p* <0.05 and *** *p* <0.001 compared with the 0 μM GM3 treated group. (**D**) After the differentiation of chondrogenic aggregates, the aggregates were digested with papain, and sulfated glycosaminoglycans and DNA content were measured. Glycosaminoglycan content was normalized to total DNA content for each sample. The values shown are the mean ± SEM from three independent experiments. * *p* < 0.05, ** *p* < 0.01 and *** *p* < 0.001 compared with the 0 μM GM3 treated group.

After chondrogenic differentiation, the hSMSC aggregates were stained with toluidine blue (for matrix proteoglycans), safranin O (for cartilage), and hematoxylin and eosin (Figure 3A–C). The 5 μM GM3 treated group had significantly increased toluidine blue (1.85 ± 0.18 folds, Figure 3A,B) and safranin O (1.80 ± 0.08 folds, Figure 3A,C) staining compared with the 0 μM treated group. Moreover, accumulation of GAG/DNA significantly increased in the 5 μM GM3 treated group compared with that in the 0 μM treated group (5 μM GM3 treated group, 16.48 ± 1.45 μg/μg; 0 μM GM3 treated group, 3.69 ± 0.12 μg/μg). Therefore, our results suggested that GM3, specifically 5 μM GM3, improved the chondrogenic differentiation of hSMSC aggregates.

### 2.3. GM3 Up-Regulated TGF-β Signaling Pathway during the Chondrogenic Differentiation of hSMSC Aggregates 

**Figure 4:**
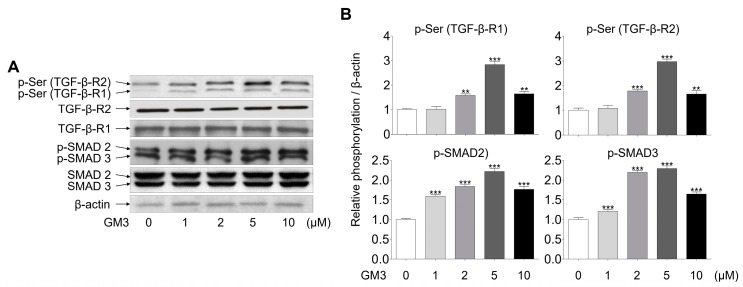
Effect of GM3 on TGF-β receptor and SMAD 2/3 activation during the chondrogenic differentiation of hSMSC aggregates. (**A**) The activation (phosphorylation) of TGF-β receptors and SMAD 2/3 was analyzed by Western blotting of chondrogenically differentiated aggregates treated with various concentrations of GM3 (0, 1, 2, 5, and 10 μM). (**B**) The data represent means ± SEM of the degree of phosphorylation observed in three separate experiments and are depicted as relative 0 μM GM3 treatment. ** *p* <0.01 and *** *p* <0.001 compared to 0 μM GM3 treatment.

Next, we investigated whether GM3 might affect TGF-β signaling, including phosphorylation of serine, TGF-β-R1, TGF-β-R2, and SMAD2/3 (downstream of TGF-β receptors). The phosphorylation of TGF-βRs, specifically TGF-β-R2, and SMAD2/3, was seen to be increased in the 5 μM GM3 treated group (Figure 4). These results indicated that GM3 and TGF-β are closely involved in the chondrogenic differentiation of hSMSC aggregates in response to TGF-βR activation. 

## 3. Discussion

MSCs provide a feasible cell source for cartilage repair because they can be differentiated to various mesodermal lineages, such as chondrocytes, osteoblasts, and adipocytes [27,28,29]. Moreover, MSCs are easy to isolate, do not have significant donor-site morbidity, and are easy to expand in vitro [30]. Notably, hSMSCs have the greatest ability for chondrogenesis than other tissue-derived MSCs because hSMSCs possess special characteristics, such as activation of uridine diphosphoglucose dehydrogenase and expression of COMP and GAG, [4,5,6,7]. Furthermore, several studies have demonstrated that hSMSCs are a valuable resource for cartilage regeneration in various animals [31,32,33]. Our results showed that hSMSCs expressed MSC specific markers and differentiated into mesodermal lineages (chondrocytes, osteoblasts, and adipocytes). Furthermore, the chondrogenically differentiated aggregates expressed chondrocyte specific markers; these characteristics make hSMSCs attractive candidates for cartilage regeneration.

Gangliosides are localized primarily on the outer cell membrane, in direct contact with the extracellular milieu, and modulate cell proliferation and differentiation [10,34]. The regulation of cell differentiation depends on various extracellular and intracellular factors. Change in ganglioside expression patterns has been observed in cells during differentiation and in response to cytokine and growth factor exposure [35,36]. Treatment gangliosides respond to several growth factors and modulate the functions of growth factor receptors such as platelet-derived growth factor receptor (PDGFR), epidermal growth factor receptor (EGFR), insulin-like growth factor receptor (IGFR), and nerve growth factor receptor (NGFR) in human and mouse stem cells [37,38,39,40,41]. Our results showed that hSMSCs did not express any gangliosides; however, chondrogenically differentiated aggregates expressed GM3 and GD3. Several studies have shown that dental pulp-derived MSCs and adipose tissue-derived MSCs expressed GM3, GM2, and GD1a [14,42]. Furthermore, GD1a expression was increased during osteoblast differentiation, and GD3 was shown to be newly expressed during neural cell differentiation [14,15,36,42]. GM3 is related to the differentiation of megakaryocytes, CD4+ T cells, CD8+ T cells, osteoblasts, and neural cells [14,15,16,36,43,44]. Interestingly, the exogenous addition of ganglioside GM3 regulates transforming growth factor-beta (TGF-β)-induced proliferation and epithelial-mesenchymal transition (EMT) [45,46,47]. Furthermore, GM3 was shown to be expressed more in normal cartilage than in osteoarthritis cartilage; specifically, ganglioside GM3 knock-out mice enhanced cartilage degradation in vivo, and led to the induction of matrix metalloproteinase (MMP-13) and ADAMTS-5 secretion and chondrocyte apoptosis in vitro; however, GM3 synthase transfection into GM3 knock-out chondrocyte suppressed MMP-13 and ADAMTS-5 expression after interleukin (IL)-1α stimulation [48,49]. Moreover, we found GD3 to be expressed in our chondrogenically differentiated aggregates. GD3 has been known to modulate inflammation; thus, we need to study the function of GD3 in chondrogenically differentiated aggregates, in the future. 

Several studies have demonstrated the role of TGF-β on cell fates, such as maturation, proliferation, and differentiation [50,51,52]. Notably, during chondrogenic differentiation, the TGF-β signal was shown to be transmitted into the nucleus through the SMAD pathway, which then activated the SOX-9 transcription factor for the expression of cartilage-specific genes, such as *type II collagen* and *aggrecan* [53]. Interestingly, TGF-β induced expression of ganglioside GM3 and GM3 synthase, and TGF-β and ganglioside GM3 treatment improved phosphorylation of TGF-β receptors and SMAD 2/3 signaling pathway [47]. Our results also showed that GM3 treatment, during the chondrogenic differentiation of hSMSC aggregates, increased the expression of chondrogenic markers. Furthermore, GM3 regulated serine phosphorylation of TGF-β-R1, TGF-β-R2, and SMAD2/3 during the chondrogenic differentiation of hSMSC aggregates. 

In conclusion, we showed that GM3 and GD3 were expressed after hSMSC aggregates were chondrogenically differentiated. Treatment with GM3—specifically with 5 μM GM3—during the chondrogenic differentiation of hSMSC aggregates, showed that the differentiated chondrogenic aggregates had a significantly increased expression of chondrogenic specific markers, higher levels of toluidine blue and safranin O staining, and higher accumulation of GAG compared with the GM3 untreated group. Furthermore, the treatment of GM3 improved the TGF-β signaling pathway via SMAD 2/3 during chondrogenic differentiation of aggregates. Taken together, our results suggested that GM3 may be useful for the development of therapeutic agents to promote cell-based articular cartilage regeneration in articular cartilage disease.

## 4. Materials and Methods

### 4.1. Chondrogenic Differentiation of hSMSCs in Aggregate Culture 

hSMSCs, isolated from human synovial tissue, were generous gifts from Dr. Yong-Gon Koh. The cells were maintained in Eagle’s alpha minimal essential medium (Thermo Fisher, Waltham, MA, USA), supplemented with 10% fetal bovine serum (Thermo Fisher, Waltham, MA, USA) and 1% penicillin-streptomycin (Thermo Fisher, Waltham, MA, USA). For the chondrogenic differentiation of aggregates, the cells were seeded at 2.5 × 10^6^ cells/mL in 15 mL polypropylene tubes and induced with 1× insulin-transferrin-selenium (Thermo Fisher, Waltham, MA, USA), 50 mM ascorbate-2-phosphate (Sigma, St. Louis, MO, USA), 100 nM dexamethasone (Sigma, St. Louis, MO, USA), and TGF-β (Peprotech, Rocky Hill, NJ, USA). The medium was replaced every 2 d for 21 d.

### 4.2. High-Performance Thin-Layer Chromatography (HPTLC)

Gangliosides extraction and purification were performed using DAEA Sephadex A25 column and Sep-Pak C18 cartridge, as previously described [14]. Also, HPTLC analysis of the gangliosides was performed using a 10 × 10 cm HPTLC 5651 plate (Merck, Frankfurter, Darmstadt, Germany), as previously described [14]. The purified gangliosides were normalized by total protein concentration and were loaded (500 μg protein/lane) onto TLC 5651 plates that were subsequently developed in chloroform/methanol/0.25% CaCl_2_·H_2_O (50:40:10, *v*/*v*/*v*) and visualized using 0.2% resorcinol. Gangliosides from murine and bovine brains were used as markers for individual ganglioside species.

### 4.3. Immunocytochemistry

Chondrogenically differentiated aggregates were fixed with 4% paraformaldehyde (PFA) for 15 min at room temperature (RT), immersed in 30% sucrose, embedded in OCT compound (Sakura, Torrance, CA, USA), and cut into 10 μm sections. Frozen sections were permeabilized with 0.1% Triton X-100 and blocked with 4% bovine serum albumin (Sigma, St. Louis, MO, USA) for 1 h at RT. The samples were incubated with primary antibodies, diluted in blocking buffer, overnight at 4 °C, washed with 0.05% Tween-20 (Sigma, St. Louis, MO, USA) in PBS, and incubated with Alexa Fluor-conjugated secondary antibodies (Thermo Fisher, Waltham, MA, USA) for 1 h at RT. Fluorescence images were captured with an Olympus microscope (Olympus, Tokyo, Japan). The antibodies used are listed in Supplementary Table S1.

### 4.4. Real-Time Polymerase Chain Reaction (PCR)

Total RNA was prepared from the samples using the TRIzol Kit (Thermo Fisher, Waltham, MA, USA), according to the manufacturer’s instructions. Reverse transcription was performed with PrimeScript first strand cDNA synthesis kit, as per the manufacturer’s instructions (Takara, Shiga, Japan). Quantitative real-time PCR was performed using the 7500 fast real-time PCR system (Applied Biosystems, Waltham, MA, USA) and fast SYBR green master mix (Applied Biosystems, Waltham, MA, USA). The primer sequences used are presented in Supplementary Table S2.

### 4.5. Histological Analysis

Frozen sections were stained with toluidine blue (to visualize matrix proteoglycans), safranin O (as an indicator of chondrogenesis), and hematoxylin and eosin, according to routine protocols. Samples were examined by light microscopy (Olympus microscope, Tokyo, Japan).

### 4.6. Measurement of GAG Content

After chondrogenic differentiation, the aggregates were digested for 18 h at 65 °C with 125 μg/mL papain in PBE buffer (10 mM EDTA and 100 mM sodium phosphate) pH 6.5, containing 5 mM L-cysteine–HCl. A total of 500 μL of enzyme preparation was used per sample. Chondrogenic potential was quantified by measuring the production of sulfated glycosaminoglycans (S-GAG) using the 1,9-dimethylmethylene blue (DMMB) assay, according to the manufacturer’s recommendations (Blyscan™ glycosaminoglycan assay kit, Biocolor, Carrickfergus, County Antrim, UK). Absorbance was measured at 656 nm using a microplate reader (Sunrise™, TECAN, Männedorf, Switzerland). 

### 4.7. Western Blot

Differentiated chondrogenic aggregates were homogenized in RIPA buffer (Sigma, St. Louis, MO, USA) and centrifuged at 15,000× *g* for 30 min. Protein concentration was measured using the Bradford method (Bio-Rad, Hercules, CA, USA). Equal amounts of protein (30 μg) were separated by 10% sodium dodecyl sulfate-polyacrylamide gel electrophoresis and then transferred to a Hybond ECL nitrocellulose membrane (Amersham Pharmacia, Amersham, Buckinghamshire, UK). The membranes were blocked in 5% skimmed milk (BD Biosciences, Franklin Lakes, NJ, USA) at RT for 2 h and then incubated with specific primary antibodies overnight, at 4 °C. The membranes were washed with TBS containing 0.1% Tween-20 and then probed with HRP-conjugated secondary antibodies. Bands were detected using SuperSignal West Femto chemiluminescent substrate (Thermo Fisher, Waltham, MA, USA). The antibodies used are listed in Supplementary Table S1.

### 4.8. Statistical Analysis

All data are presented as mean ± SEM. Comparison of multiple groups was performed by one-way analysis of variance (ANOVA), followed by pairwise comparison with the Bonferroni post-hoc test. All data were analyzed using GraphPad Prism version 5.00 software (GraphPad Software, San Diego, CA, USA).

## Supplementary Materials

Supplementary materials can be found at https://www.mdpi.com/1422-0067/21/6/1967/s1.

## References

[1] Dominici M., Le Blanc K., Mueller I., Slaper-Cortenbach I., Marini F., Krause D., Deans R., Keating A., Prockop D., Horwitz E. (2006). Minimal criteria for defining multipotent mesenchymal stromal cells. The International Society for Cellular Therapy position statement. Cytotherapy.

[2] Pittenger M.F., Mackay A.M., Beck S.C., Jaiswal R.K., Douglas R., Mosca J.D., Moorman M.A., Simonetti D.W., Craig S., Marshak D.R. (1999). Multilineage potential of adult human mesenchymal stem cells. Science.

[3] Sakaguchi Y., Sekiya I., Yagishita K., Muneta T. (2005). Comparison of human stem cells derived from various mesenchymal tissues: superiority of synovium as a cell source. Arthritis Rheum..

[4] Wilkinson L.S., Moore A.R., Pitsillides A.A., Willoughby D.A., Edwards J.C. (1993). Comparison of surface fibroblastic cells in subcutaneous air pouch and synovial lining: differences in uridine diphosphoglucose dehydrogenase activity. Int. J. Exp. Pathol..

[5] Recklies A.D., Baillargeon L., White C. (1998). Regulation of cartilage oligomeric matrix protein synthesis in human synovial cells and articular chondrocytes. Arthritis Rheum..

[6] Fife R.S., Caterson B., Myers S.L. (1985). Identification of link proteins in canine synovial cell cultures and canine articular cartilage. J. Cell Biol..

[7] Hamerman D., Smith C., Keiser H.D., Craig R. (1982). Glycosaminoglycans produced by human synovial cell cultures. Coll. Relat. Res..

[8] Hakomori S. (1990). Bifunctional role of glycosphingolipids. Modulators for transmembrane signaling and mediators for cellular interactions. J. Biol. Chem..

[9] Hakomori S., Yamamura S., Handa A.K. (1998). Signal transduction through glyco(sphingo)lipids. Introduction and recent studies on glyco(sphingo)lipid-enriched microdomains. Ann. N. Y. Acad. Sci..

[10] Hakomori S. (1981). Glycosphingolipids in cellular interaction, differentiation, and oncogenesis. Annu. Rev. Biochem..

[11] Hakomori S. (2002). Glycosylation defining cancer malignancy: new wine in an old bottle. Proc. Natl. Acad. Sci. USA.

[12] Yu R.K., Macala L.J., Taki T., Weinfield H.M., Yu F.S. (1988). Developmental changes in ganglioside composition and synthesis in embryonic rat brain. J. Neurochem..

[13] Yu R.K. (1994). Development regulation of ganglioside metabolism. Prog. Brain Res..

[14] Ryu J.S., Ko K., Lee J.W., Park S.B., Byun S.J., Jeong E.J., Ko K., Choo Y.K. (2009). Gangliosides are involved in neural differentiation of human dental pulp-derived stem cells. Biochem. Biophys. Res. Commun..

[15] Yang H.J., Jung K.Y., Kwak D.H., Lee S.H., Ryu J.S., Kim J.S., Chang K.T., Lee J.W., Choo Y.K. (2011). Inhibition of ganglioside GD1a synthesis suppresses the differentiation of human mesenchymal stem cells into osteoblasts. Dev. Growth Differ..

[16] Lee D.H., Koo D.B., Ko K., Ko K., Kim S.M., Jung J.U., Ryu J.S., Jin J.W., Yang H.J., Do S.I. (2007). Effects of daunorubicin on ganglioside expression and neuronal differentiation of mouse embryonic stem cells. Biochem. Biophys. Res. Commun..

[17] Chung T.W., Kim S.J., Choi H.J., Kim K.J., Kim M.J., Kim S.H., Lee H.J., Ko J.H., Lee Y.C., Suzuki A. (2009). Ganglioside GM3 inhibits VEGF/VEGFR-2-mediated angiogenesis: direct interaction of GM3 with VEGFR-2. Glycobiology.

[18] Kabayama K., Sato T., Saito K., Loberto N., Prinetti A., Sonnino S., Kinjo M., Igarashi Y., Inokuchi J. (2007). Dissociation of the insulin receptor and caveolin-1 complex by ganglioside GM3 in the state of insulin resistance. Proc. Natl. Acad. Sci. USA.

[19] Kawashima N., Yoon S.J., Itoh K., Nakayama K. (2009). Tyrosine kinase activity of epidermal growth factor receptor is regulated by GM3 binding through carbohydrate to carbohydrate interactions. J. Biol. Chem..

[20] Kim Y.I., Ryu J.S., Yeo J.E., Choi Y.J., Kim Y.S., Ko K., Koh Y.G. (2014). Overexpression of TGF-beta1 enhances chondrogenic differentiation and proliferation of human synovium-derived stem cells. Biochem. Biophys. Res. Commun..

[21] Hao J., Varshney R.R., Wang D.A. (2009). Engineering osteogenesis and chondrogenesis with gene-enhanced therapeutic cells. Curr. Opin. Mol. Ther..

[22] Park J.S., Woo D.G., Yang H.N., Lim H.J., Chung H.M., Park K.H. (2008). Heparin-bound transforming growth factor-beta3 enhances neocartilage formation by rabbit mesenchymal stem cells. Transplantation.

[23] Chimal-Monroy J., Diaz de Leon L. (1999). Expression of N-cadherin, N-CAM, fibronectin and tenascin is stimulated by TGF-beta1, beta2, beta3 and beta5 during the formation of precartilage condensations. Int. J. Dev. Biol..

[24] Heldin C.H., Miyazono K., ten Dijke P. (1997). TGF-beta signalling from cell membrane to nucleus through SMAD proteins. Nature.

[25] Saika S., Kono-Saika S., Ohnishi Y., Sato M., Muragaki Y., Ooshima A., Flanders K.C., Yoo J., Anzano M., Liu C.Y. (2004). Smad3 signaling is required for epithelial-mesenchymal transition of lens epithelium after injury. Am. J. Pathol..

[26] Derynck R., Zhang Y.E. (2003). Smad-dependent and Smad-independent pathways in TGF-beta family signalling. Nature.

[27] Sekiya I., Vuoristo J.T., Larson B.L., Prockop D.J. (2002). In vitro cartilage formation by human adult stem cells from bone marrow stroma defines the sequence of cellular and molecular events during chondrogenesis. Proc. Natl. Acad. Sci. USA.

[28] Sekiya I., Larson B.L., Vuoristo J.T., Cui J.G., Prockop D.J. (2004). Adipogenic differentiation of human adult stem cells from bone marrow stroma (MSCs). J. Bone Miner. Res..

[29] Horwitz E.M., Prockop D.J., Fitzpatrick L.A., Koo W.W., Gordon P.L., Neel M., Sussman M., Orchard P., Marx J.C., Pyeritz R.E. (1999). Transplantability and therapeutic effects of bone marrow-derived mesenchymal cells in children with osteogenesis imperfecta. Nat. Med..

[30] Jones E.A., Kinsey S.E., English A., Jones R.A., Straszynski L., Meredith D.M., Markham A.F., Jack A., Emery P., McGonagle D. (2002). Isolation and characterization of bone marrow multipotential mesenchymal progenitor cells. Arthritis Rheum..

[31] Nakagawa Y., Muneta T., Kondo S., Mizuno M., Takakuda K., Ichinose S., Tabuchi T., Koga H., Tsuji K., Sekiya I. (2015). Synovial mesenchymal stem cells promote healing after meniscal repair in microminipigs. Osteoarthr. Cartil..

[32] Hatsushika D., Muneta T., Nakamura T., Horie M., Koga H., Nakagawa Y., Tsuji K., Hishikawa S., Kobayashi E., Sekiya I. (2014). Repetitive allogeneic intraarticular injections of synovial mesenchymal stem cells promote meniscus regeneration in a porcine massive meniscus defect model. Osteoarthr. Cartil..

[33] Katagiri H., Muneta T., Tsuji K., Horie M., Koga H., Ozeki N., Kobayashi E., Sekiya I. (2013). Transplantation of aggregates of synovial mesenchymal stem cells regenerates meniscus more effectively in a rat massive meniscal defect. Biochem. Biophys. Res. Commun..

[34] Yanagisawa M. (2011). Stem cell glycolipids. Neurochem. Res..

[35] Liu Y., Li R., Ladisch S. (2004). Exogenous ganglioside GD1a enhances epidermal growth factor receptor binding and dimerization. J. Biol. Chem..

[36] Kim S.M., Jung J.U., Ryu J.S., Jin J.W., Yang H.J., Ko K., You H.K., Jung K.Y., Choo Y.K. (2008). Effects of gangliosides on the differentiation of human mesenchymal stem cells into osteoblasts by modulating epidermal growth factor receptors. Biochem. Biophys. Res. Commun..

[37] Giordano F., De Marzo A., Vetrini F., Marigo V. (2007). Fibroblast growth factor and epidermal growth factor differently affect differentiation of murine retinal stem cells in vitro. Mol. Vis..

[38] Vrijens P., Noppen S., Boogaerts T., Vanstreels E., Ronca R., Chiodelli P., Laporte M., Vanderlinden E., Liekens S., Stevaert A. (2019). Influenza virus entry via the GM3 ganglioside-mediated platelet-derived growth factor receptor beta signalling pathway. J. Gen. Virol..

[39] Dam D.H.M., Wang X.Q., Sheu S., Vijay M., Shipp D., Miller L., Paller A.S. (2017). Ganglioside GM3 Mediates Glucose-Induced Suppression of IGF-1 Receptor-Rac1 Activation to Inhibit Keratinocyte Motility. J. Investig. Dermatol..

[40] Li Y., Huang X., Wang C., Li Y., Luan M., Ma K. (2015). Ganglioside GM3 exerts opposite effects on motility via epidermal growth factor receptor and hepatocyte growth factor receptor-mediated migration signaling. Mol. Med. Rep..

[41] Wang X.Q., Sun P., Go L., Koti V., Fliman M., Paller A.S. (2006). Ganglioside GM3 promotes carcinoma cell proliferation via urokinase plasminogen activator-induced extracellular signal-regulated kinase-independent p70S6 kinase signaling. J. Investig. Dermatol..

[42] Lee S.H., Ryu J.S., Lee J.W., Kwak D.H., Ko K., Choo Y.K. (2010). Comparison of ganglioside expression between human adipose- and dental pulp-derived stem cell differentiation into osteoblasts. Arch. Pharm. Res..

[43] Jin U.H., Chung T.W., Song K.H., Kwak C.H., Choi H.J., Ha K.T., Chang Y.C., Lee Y.C., Kim C.H. (2014). Ganglioside GM3 is required for caffeic acid phenethyl ester-induced megakaryocytic differentiation of human chronic myelogenous leukemia K562 cells. Biochem. Cell Biol..

[44] Nagafuku M., Okuyama K., Onimaru Y., Suzuki A., Odagiri Y., Yamashita T., Iwasaki K., Fujiwara M., Takayanagi M., Ohno I. (2012). CD4 and CD8 T cells require different membrane gangliosides for activation. Proc. Natl. Acad. Sci. USA.

[45] Kwak D.H., Lee S., Kim S.J., Ahn S.H., Song J.H., Choo Y.K., Choi B.K., Jung K.Y. (2005). Ganglioside GM3 inhibits the high glucose- and TGF-beta1-induced proliferation of rat glomerular mesangial cells. Life Sci..

[46] Guan F., Handa K., Hakomori S.I. (2009). Specific glycosphingolipids mediate epithelial-to-mesenchymal transition of human and mouse epithelial cell lines. Proc. Natl. Acad. Sci. USA.

[47] Kim S.J., Chung T.W., Choi H.J., Kwak C.H., Song K.H., Suh S.J., Kwon K.M., Chang Y.C., Park Y.G., Chang H.W. (2013). Ganglioside GM3 participates in the TGF-beta1-induced epithelial-mesenchymal transition of human lens epithelial cells. Biochem. J..

[48] David M.J., Portoukalian J., Rebbaa A., Vignon E., Carret J.P., Richard M. (1993). Characterization of gangliosides from normal and osteoarthritic human articular cartilage. Arthritis Rheum..

[49] Sasazawa F., Onodera T., Yamashita T., Seito N., Tsukuda Y., Fujitani N., Shinohara Y., Iwasaki N. (2014). Depletion of gangliosides enhances cartilage degradation in mice. Osteoarthr. Cartil..

[50] Debacq-Chainiaux F., Borlon C., Pascal T., Royer V., Eliaers F., Ninane N., Carrard G., Friguet B., de Longueville F., Boffe S. (2005). Repeated exposure of human skin fibroblasts to UVB at subcytotoxic level triggers premature senescence through the TGF-beta1 signaling pathway. J. Cell Sci..

[51] Jian H., Shen X., Liu I., Semenov M., He X., Wang X.F. (2006). Smad3-dependent nuclear translocation of beta-catenin is required for TGF-beta1-induced proliferation of bone marrow-derived adult human mesenchymal stem cells. Genes Dev..

[52] Pei M., He F., Vunjak-Novakovic G. (2008). Synovium-derived stem cell-based chondrogenesis. Differentiation.

[53] Kulyk W.M., Franklin J.L., Hoffman L.M. (2000). Sox9 expression during chondrogenesis in micromass cultures of embryonic limb mesenchyme. Exp. Cell Res..

