# Supplementary material for: Ganglioside GM3 Up-Regulate Chondrogenic Differentiation by Transform Growth Factor Receptors
Source: Int J Mol Sci. 2020 Mar 13;21(6):1967. doi: 10.3390/ijms21061967 (PMC7139639; doi:10.3390/ijms21061967)
Supplement: Supplementary file 1 [file ijms-21-01967-s001.pdf]

Supplementary materials and methods

FACS analysis

After trypsinization, hSMSCs were resuspended in ice cold phosphate buffered saline, containing 0.5% bovine serum albumin, at a concentration of 1.0 × 10^6 cells/mL. Thereafter, the cells were incubated for 20 min on ice with PerCP-conjugated anti-human CD4, PE-conjugated anti-human CD34, APC-conjugated anti-human CD45, PE-conjugated anti-human CD44, FITC-conjugated anti-human CD73, PerCP-conjugated anti-human CD90, or PerCP-conjugated anti-human CD105 (BD Biosciences, Franklin Lakes, NJ, USA). Analyses were performed by flow cytometry (BD Biosciences, Franklin Lakes, NJ, USA) using the CellQuest software (BD Biosciences, Franklin Lakes, NJ, USA).

Chondrocyte differentiation

hSMSCs were seeded at 5 × 10^4 cells/well in 24-well tissue culture plates and grown in standard growth media for 1 d. The medium was replaced with chondrogenic differentiation media, consisting of high-glucose DMEM with 1× Insulin-Transferrin-Selenium (Thermo Fisher, Waltham, MA, USA), 50 mM ascorbate-2-phosphate (Sigma, St. Louis, MO, USA), 100 nM dexamethasone (Sigma, St. Louis, MO, USA), and TGF- β (Peprotech , Rocky Hill, NJ, USA). The medium was replaced every 2 d for 21 d. Post-differentiation, the cultured cells were fixed in 4% PFA and stained with toluidine blue (Sigma, St. Louis, MO, USA). Samples were examined by light microscopy (Olympus, Tokyo, Japan).

Osteoblast differentiation

hSMSCs were seeded at 5 × 10^4 cells/well in 24-well tissue culture plates and grown in standard growth media for 1 d. The medium was replaced with osteogenic differentiation media consisting of high-glucose DMEM with 1× penicillin/streptomycin, 10% fetal bovine serum, 100 nM dexamethasone, 10 mM β-glycerophosphate (Sigma, St. Louis, MO, USA), and 50 μM ascorbic acid for 21 d. The medium was changed every 2 d. After differentiation, the cultured cells were fixed in 4% PFA and stained with fresh 2% alizarin red S pH 7.2 (Sigma, St. Louis, MO, USA). Samples were examined by light microscopy (Olympus, Tokyo, Japan).

Adipocyte differentiation

hSMSCs were seeded at 5 × 10^3 cells/well in 12-well tissue culture plates and grown in standard growth media for 1 d. The medium was replaced with adipogenic differentiation media, which consisted of complete high-glucose DMEM with 1× penicillin/streptomycin, 10% fetal bovine serum, 1 μM dexamethasone, 200 μM indomethacin (Sigma, St. Louis, MO, USA), 0.5 mM 3-butyl-1-methylxanthine (IBMX) (Sigma, St. Louis, MO, USA), and 0.01 mg/mL insulin (GIBCO, Waltham, MA, USA) for 21 d. The medium was exchanged every 2 d. After differentiation, the cultured cells were fixed in 4% PFA and stained with fresh oil red O solution (Sigma, St. Louis, MO, USA). Samples were examined *via* light microscopy (Olympus, Tokyo, Japan).

Cell viability assay

hSMSCs (4 × 10^4 cells/well) were cultured in 96-well microtiter plates under CO_2 in a humidified atmosphere for 24 h at 37 °C. After cell culturing, GM3 (Matreya, State College, PA, USA) was added to each well at a given concentration (0, 1, 2, 5, and 10 μM). Each well was then incubated in CCK-8 (Dojindo, Kumamoto, JAPAN) solution for 2 h. The absorbance of each well was determined at 450 nm wavelength by a spectrophotometer.

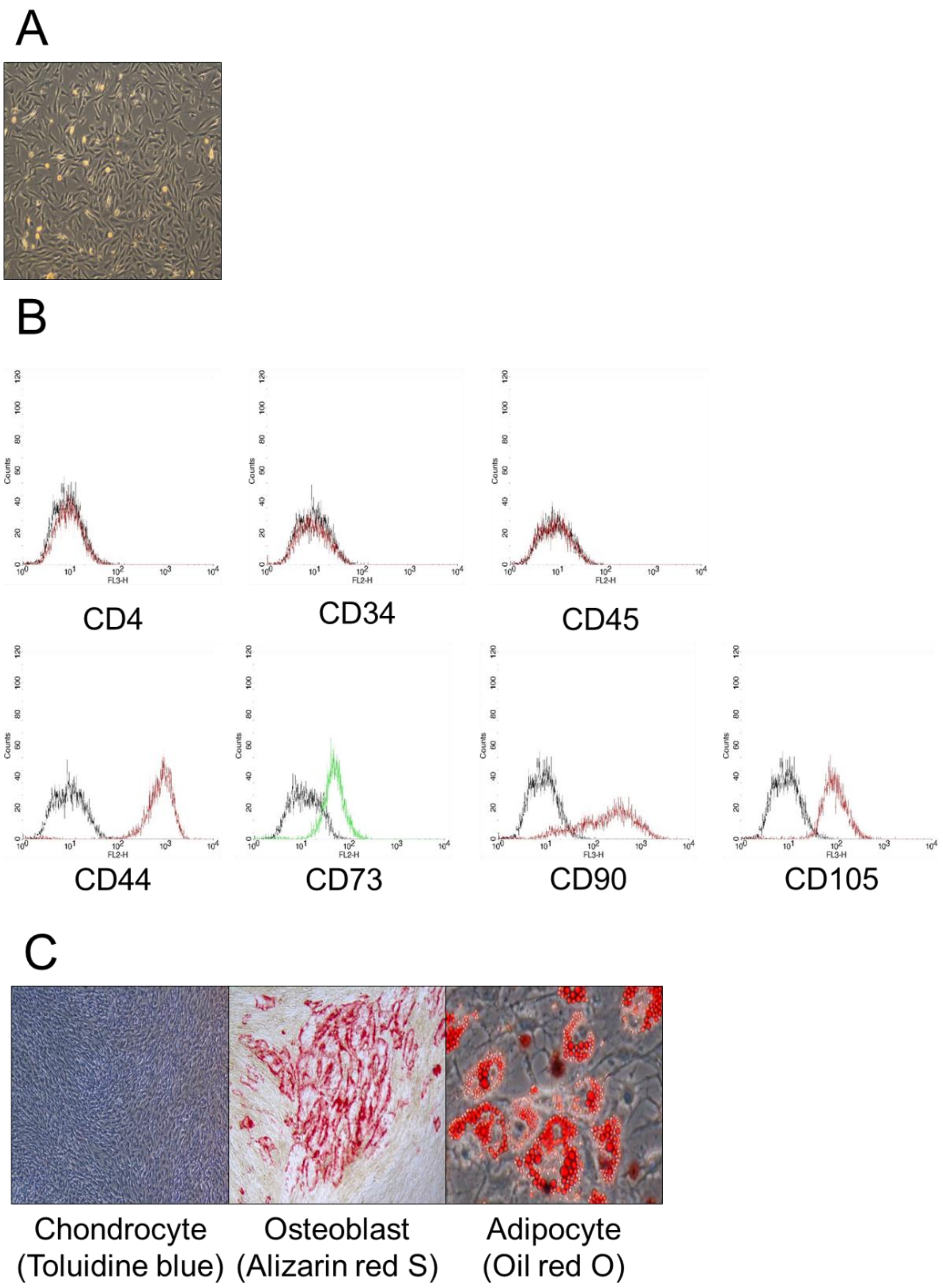

Figure S1. Characterization of human synovium-derived mesenchymal stem cells (hSMSCs). (A) Morphology of hSMSCs established from human synovial tissue (B) FACS analysis for expression of MSC negative surface proteins, CD4, CD34, and CD45 (upper), and positive surface proteins, CD44, CD73, CD90, and CD105 (bottom). (C) Analysis of differentiation capacity. Chondrocytes (left, toluidine blue), osteoblast (middle, alizarin red S), and adipocyte (right, oil red O) from hSMSCs.

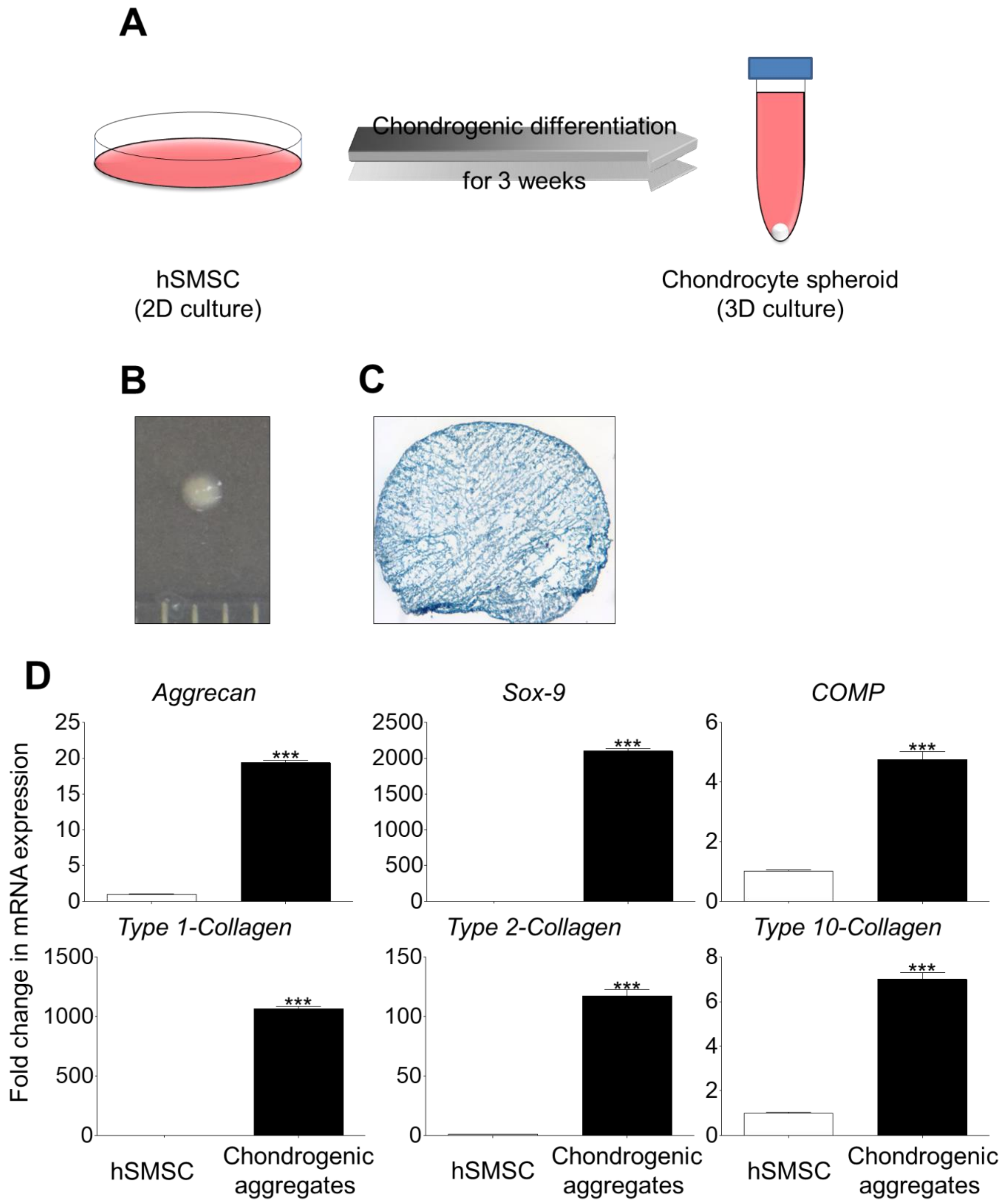

Figure S2. Chondrogenic differentiation of aggregates. (A) Scheme of chondrogenic differentiation hSMSCs aggregates. (B) Representative microscopic images of differentiated chondrogenic aggregates after 21 d of culture in differentiation medium. (C) Staining with toluidine blue for differentiated chondrogenic aggregates after 21 d of culture in differentiation medium. (D) mRNA expression of chondrogenic specific markers after chondrogenic differentiation of aggregates. mRNA expression levels were normalized to the housekeeping gene β-actin. The values shown are the means ± SEM from three independent experiments. ***$p < 0.001$ compared with hSMSCs.

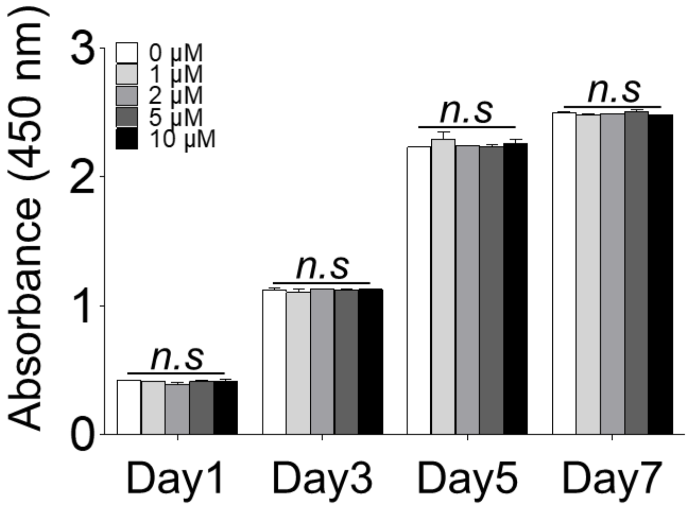

Figure S3. Effect of GM3 on cell viability in hSMSCs. hSMSCs were treated with ganglioside GM3 (0, 1, 2, 5, and 10 μM) for 7 d. The values shown are the means ± SEM from six independent experiments.

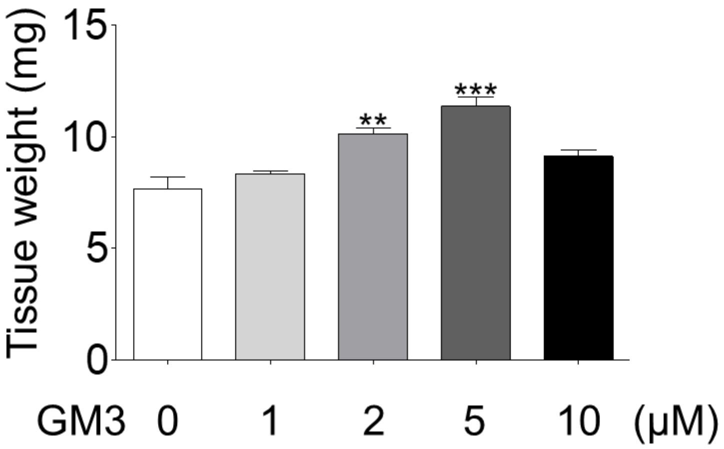

Figure S4. Effect of GM3 on differentiated chondrogenic aggregates tissue weight. Analysis of chondrogenically differentiated aggregate tissue weight at 21 d after chondrocyte differentiation. The values shown are the means ± SEM from six independent experiments. **$p < 0.01$ and ***$p < 0.001$ compared with the 0 μM GM3 treated group.

Supplementary Table 1. List of antibodies used in this study.

Antibodies	Catalog No.	Company	Dilution
anti-phospho-serine	sc-81514	SantaCruz Biotechnology	1:200
anti-TGF-β receptor 2	sc-17792	SantaCruz Biotechnology	1:1000
anti-TGF-β receptor 1	sc-518018	SantaCruz Biotechnology	1:1000
anti-phospho-SMAD 2/3	#8828	Cell Signal Technology	1:1000
anti-SMAD 2/3	#8685	Cell Signal Technology	1:1000
anti-β-actin	sc-47778	SantaCruz Biotechnology	1:1000
Anti-ganglioside GM3	370695	Seikagaku	1:250
anti-Mouse-HRP	sc-2005	SantaCruz Biotechnology	1:1000
anti-Rabbit-HRP	sc-2004	SantaCruz Biotechnology	1:1000
anti-Mouse-Alexa488	A32723	Thermo Fisher	1:200

Supplementary Table 2. List of primers used in this study.

Gene	Primer (Forward)	Primer (Reverse)
GM3 synthase	AGGAATGTCGTCCCAAGTTTG	GGAGTAAGTCCACGCTATACCT
Aggrecan	CTGAGTGAAACCACCTCTGCATT	GACGCCTCGCCTTCTTGA
SOX-9	CCCCAACAGATCGCCTACAG	TCTGGTGGTCGGTGTAGTCGTA
COMP	CAAGAAGTCCTATCGTTGGTTCCT	CTCAGGGCCCTCATAGAATCG
Type 1 collagen	GTGCGATGACGTGATCTGTGA	CGGTGGTTTCTTGGTCGGT
Type 2 collagen	GGCAATAGCAGGTTCACGTACA	CGATAACAGTCTTGCCCCACTT
Type 10 collagen	ATGCTGCCACAAATACCCTTT	GGTAGTGGGCCTTTTATGCCT
β-actin	GGACTTCGAGCAAGAGATGG	AGCACTGTGTTGGCGTACAG
